# Supplementary material for: Unveiling the Nutritional Quality of Terrestrial Animal Source Foods by Species and Characteristics of Livestock Systems
Source: Nutrients. 2024 Oct 2;16(19):3346. doi: 10.3390/nu16193346 (PMC11478523; doi:10.3390/nu16193346)
Supplement: Supplementary file 1 [file nutrients-16-03346-s001.zip › nutrients-3162492-supplementary.pdf]

Supplementary Materials

Table S1. Search terms used in the review by database 1

Table S2. Food composition databases of poultry eggs....................2

Table S3. Food composition databases of milk from mammalian livestock species and humans 3

Table S4. Food composition databases of meat from mammalian and avian livestock species 4

Table S1. Search terms used in the review by database

**Search terms using Academic Search Complete via EBSCOHost**
(“animal source food” OR “livestock derived food” OR “animal derived food” OR meat OR “red meat” OR “dairy product”) AND (nutrient OR “nutritional composition” OR “nutrient composition” OR “nutritional content” OR macronutrient OR micronutrient OR “nutritional value”)
**Search terms using PubMed**
(“animal source food” OR “animal sourced food” OR “animal based food” OR “livestock derived food” OR TASF) AND (macronutrient OR protein OR amino acid OR lipid OR “DHA” OR “EPA” OR carbohydrate OR “protein quality” OR micronutrient OR “vitamin A” OR calcium OR iron OR “bioactive compound” OR taurine OR carnosine OR creatine) OR ((eggs AND (poultry OR duck OR emu OR “muscovy duck” OR ostrich OR partridge OR peafowl OR pheasant OR “pig” OR pigeon)) OR ((milk OR “dairy product”) AND (“lactose” OR buffalo OR camel OR cow OR cattle OR donkey OR goat OR mare OR mithan OR sheep)) OR ((“red meat” OR meat) AND (alpaca OR beef OR goat OR guinea pig OR llama OR pheasant OR pig OR poultry OR sheep)) OR ((Insect OR honey OR “bee products” OR “apis” OR “bees” OR “game meat” OR “bushmeat” OR “wildlife food”) AND (“nutrient composition” OR “nutrient value” OR “nutritional value” OR “nutrient density” OR “nutrient content”)) NOT (((“aquatic animal food” OR “fortified”) AND “animal source food”) OR (ingredient” AND “animal source food”))
**Search terms using ScienceDirect**
(“animal source food” OR “animal sourced food” OR “animal based food” OR “livestock derived food” OR TASF) AND (macronutrient OR protein OR amino acid OR lipid OR “DHA” OR “EPA” OR carbohydrate OR “protein quality” OR micronutrient OR “vitamin A” OR calcium OR iron OR “bioactive compound” OR taurine OR carnosine OR creatine) OR ((eggs AND (poultry OR duck OR emu OR “muscovy duck” OR ostrich OR partridge OR peafowl OR pheasant OR “pig” OR pigeon)) OR ((milk OR “dairy product”) AND (“lactose” OR buffalo OR camel OR cow OR cattle OR donkey OR goat OR mare OR mithan OR sheep)) OR ((“red meat” OR meat) AND (alpaca OR beef OR goat OR guinea pig OR llama OR pheasant OR pig OR poultry OR sheep)) OR ((Insect OR honey OR “bee products” OR “apis” OR “bees” OR “game meat” OR “bushmeat” OR “wildlife food”) AND (“nutrient composition” OR “nutrient value” OR “nutritional value” OR “nutrient density” OR “nutrient content”)) NOT (((“aquatic animal food” OR “fortified”) AND “animal source food”) OR (ingredient” AND “animal source food”))

Table S2. Food composition databases of poultry eggs

Animal species	Food Composition Database/Source	Description	Energy (kcal)	Energy (kJ)	Protein (g)	Fat (g)	Carbohydrates (g)	Vitamin A (µg) RAE*	Riboflavin (mg)	Vitamin B12 (µg)	Calcium (mg)	Iron (mg)	Zinc (mg)
Chicken	Australia and New Zealand	Egg, chicken, whole, raw	127	533	12.6	8.5	0.3	130	0.43	1.40	47	1.90	1.1
	USA	Egg, whole, raw, fresh	143	599	12.6	9.51	0.7	160	0.46	0.89	56	1.75	1.3
	Western Africa	Egg, chicken, raw	150	626	13.5	10.6	0.2	122	0.51	1.40	57	2.20	1.5
Average			140.00	586.00	13.05	9.55	0.41	137.33	0.47	1.23	53.33	1.95	1.31
Standard deviation			11.79	47.84	0.64	1.48	0.28	20.03	0.04	0.29	5.51	0.23	0.19
Egg yolk	Australia and New Zealand	Egg, chicken, yolk, raw	313	1311	15.6	28.2	0.2	449	0.40	4.00	100	4.00	2.5
	USA	Egg, yolk, raw, fresh	322	1350	15.9	26.5	3.6	381	0.53	1.95	129	2.73	2.3
Average			317.50	1330.50	15.75	27.35	1.90	415.00	0.46	2.98	114.50	3.37	2.40
Standard deviation			6.36	27.58	0.21	1.20	2.40	48.08	0.09	1.45	20.51	0.90	0.14
Egg white	Australia and New Zealand	Egg, chicken, white (albumen), raw	47	197	11.2	0.0	0.4	0	0.41	0.00	5	0.20	0.0
	USA	Egg, white, raw, fresh	52	216	10.9	0.2	0.7	0	0.44	0.09	7	0.08	0.0
Average			49.50	206.50	11.05	0.09	0.57	0.00	0.42	0.05	6.00	0.14	0.02
Standard deviation			3.54	13.44	0.21	0.12	0.23	0.00	0.02	0.06	1.41	0.08	0.02
Turkey	USA	Egg, turkey, whole, fresh, raw	171	716	13.7	11.9	1.2	166	0.47	1.69	99	4.10	1.6
	Western Africa	Egg, turkey, raw	184	764	11.5	13.0	5.1	80	0.05	2.20	99	6.30	2.6
Average			177.50	740.00	12.60	12.45	3.13	123.00	0.26	1.95	99.00	5.20	2.09
Standard deviation			9.19	33.94	1.56	0.78	2.79	60.81	0.30	0.36	0.00	1.56	0.72
Quail	USA	Egg, quail, whole, fresh, raw	158	663	13.0	11.1	0.4	156	0.79	1.58	64	3.65	1.5
	Western Africa	Egg, quail, raw	180	751	15.6	11.9	2.7	42	0.07	2.00	81	5.30	2.2
Average			169.00	707.00	14.30	11.50	1.56	99.00	0.43	1.79	72.50	4.48	1.84
Standard deviation			15.56	62.23	1.84	0.57	1.62	80.61	0.51	0.30	12.02	1.17	0.52
Duck	ASEAN	Egg, duck, whole	183	766	12.6	13.6	2.5	328	0.38	nd	62	3.20	1.0
	USA	Egg, duck, whole, fresh, raw	185	776	12.8	13.8	1.5	194	0.40	5.40	64	3.85	1.4
	Western Africa	Egg, duck, raw	179	742	11.8	13.6	2.4	184	0.07	5.40	52	2.50	1.5
Average			182.33	761.33	12.40	13.67	2.12	235.33	0.28	5.40	59.33	3.18	1.30
Standard deviation			3.06	17.47	0.53	0.12	0.58	80.41	0.19	0.00	6.43	0.68	0.26

Geese	USA	Egg, goose, whole, fresh, raw	185	775	13.9	13.3	1.4	187	0.38	5.10	60	3.64	1.3

Table S3. Food composition databases of milk from mammalian livestock species and humans

Animal species	Food Composition Database/Source	Description	Energy (kcal)	Energy (kJ)	Protein (g)	Fat (g)	Carbohydrates (g)	Vitamin A (µg) RAE*	Riboflavin (mg)	Vitamin B12 (µg)	Calcium (mg)	Iron (mg)	Zinc (mg)
Human milk	Australia and New Zealand	Milk, human/breast, mature, fluid	71	298	1.4	4.4	7.1	62	0.04	0.10	33.00	0.03	0.2
	Colombia	Leche humana, madura, líquida	71	294	1.1	4.3	7.0	64	0.04	0.05	32.00	0.00	0.2
	Denmark	Human milk, mature	71	296	1.3	4.1	7.2	60	0.03	0.00	34.00	0.07	0.3
	USA	Milk, human, mature, fluid	70	291	1.0	4.4	6.9	61	0.04	0.05	32.00	0.03	0.2
	Western Africa	Breastmilk mature	70	292	1.1	3.5	8.4	61	0.04	0.05	26.00	0.20	0.2
Average			70.60	294.20	1.19	4.14	7.32	61.60	0.04	0.05	31.40	0.07	0.21
Standard deviation			0.55	2.86	0.16	0.37	0.62	1.52	0.00	0.04	3.13	0.08	0.05
Buffalo [Average]	Medhammar et al., 2011		99	412	4.0	7.5	nd	69	0.11	0.45	191.00	0.17	0.5
Cow	ASEAN	Milk, cow, fresh	68	285	3.5	3.5	4.2	35	0.19	-	122.00	0.30	-
	Australia and New Zealand	Milk, cow, fluid, regular fat (3.5% fat)	69	290	3.5	3.5	6.1	50	0.21	0.60	107.00	0.03	0.4
	Colombia	Leche de vaca entera líquida cruda	65	271	3.3	3.7	4.6	43	0.18	0.36	120.00	0.20	0.8
	Denmark	Milk, whole, konventional (not organic), 3.5 % fat	63	264	3.4	3.5	4.6	32	0.17	0.45	116.00	0.02	0.4
	USA	Milk, whole, 3.25% milkfat, without added vitamin A and vitamin D	61	256	3.2	3.3	4.8	46	0.17	0.45	113.00	0.03	0.4
	Western Africa	Milk, cow, whole, pasteurized or UHT, 3.5% fat	64	270	3.3	3.4	5.1	42	0.27	0.59	119.00	0.10	0.6
Average			65.07	272.63	3.37	3.48	4.90	41.28	0.20	0.49	116.17	0.11	0.51
Standard deviation			3.01	12.78	0.12	0.14	0.66	6.83	0.04	0.10	5.49	0.11	0.20
Mithan [Average]	Medhammar et al., 2011		122	510	6.5	8.9	nd	nd	nd	nd	88.00	nd	nd
Yak [Average]	Medhammar et al., 2011		100	417	5.2	6.8	nd	nd	nd	nd	129.00	0.57	0.9
Goat	ASEAN	Milk, goat, fluid, full cream	71	297	3.6	4.1	4.8	35	0.04	-	136.00	0.40	-

	Colombia	Leche de cabra entera liquida cruda	74	308	3.9	4.5	4.4	24	0.14	0.07	120.00	0.10	0.4
	Denmark	Goat milk	69	289	3.6	4.1	4.4	35	0.14	0.07	134.00	0.05	0.3
	USA	Milk, goat, fluid, with added vitamin D	69	288	3.6	4.1	4.5	57	0.14	0.07	134.00	0.05	0.3
	Western Africa	Milk goat fresh	83	345	3.9	5.3	4.9	37	0.17	0.10	159.00	0.10	0.4
Average			73.20	305.40	3.71	4.43	4.59	37.64	0.13	0.08	136.60	0.14	0.35
Standard deviation			5.85	23.54	0.17	0.52	0.24	11.98	0.05	0.01	14.06	0.15	0.05
Sheep	USA	Milk, sheep, fluid. USDA 2019:	108	451	6.0	7.0	5.4	44	0.36	0.71	193.00	0.10	0.5
	Western Africa	Milk sheep fresh	100	416	5.9	6.4	4.7	45	0.18	0.50	164.00	0.10	0.6
Average			104.00	433.50	5.94	6.70	5.03	44.50	0.27	0.61	178.50	0.10	0.58
Standard deviation			5.66	24.75	0.06	0.42	0.47	0.71	0.12	0.15	20.51	0.00	0.06
Alpaca [Average]	Medhammar et al., 2011		71	299	5.8	3.2	5.1	nd	nd	nd	nd	nd	nd
Bactrian Camel [Average]	Medhammar et al., 2011		76	319	3.9	5.0	4.2	97	0.12	nd	153.70	nd	0.7
Dromedary Camel [Average]	Medhammar et al., 2011		56	234	3.1	3.2	nd	nd	0.06	nd	114.00	0.21	0.6
Llama [Average]	Medhammar et al., 2011		78	326	4.1	4.2	nd	nd	nd	nd	195.00	nd	1.1
Reideer [Average]	Medhammar et al., 2011		196	819	10.4	16.1	nd	nd	nd	nd	320.00	nd	1.1
Donkey [Average]	Medhammar et al., 2011		37	156	1.6	0.7	nd	nd	0.03	nd	91.00	nd	nd
Mare [Average]	Medhammar et al., 2011		48	199	2.0	1.6	nd	nd	0.02	nd	95.00	0.10	0.2

Table S4. Food composition databases of meat from mammalian and avian livestock species

Animal species	Food Composition Database/Source	Description	Energy (kcal)	Energy (kJ)	Protein (g)	Fat (g)	Carbohydrates (g)	Vitamin A (µg) RAE*	Riboflavin (mg)	Vitamin B12 (µg)	Calcium (mg)	Iron (mg)	Zinc (mg)
Cattle (Beef)	Australia and New Zealand	Beef, all cuts, separable fat, raw	592	2478	12.10	61.40	0.00	0	0.05	2.90	6	1.30	0.9
	USA	Beef, variety meats and by-products, mechanically separated beef, raw	276	1160	15.00	23.50	0.00	0	0.12	2.56	485	5.67	3.6

	Western Africa	Beef meat, lean ca, 5% fat raw	131	525	22.50	4.60	0.00	6	0.15	2.40	18	2.40	3.5
Average			333.00	1387.67	16.53	29.83	0.00	2.01	0.11	2.62	169.67	3.12	2.69
Standard deviation			235.73	996.21	5.37	28.92	0.00	3.45	0.05	0.26	273.15	2.27	1.51
Water buffalo (Carabeef)	USA	Game meat, buffalo, water, raw	99	414	20.40	1.40	0.00	0	0.20	1.66	12	1.61	1.9
Sheep	ASEAN	Mutton lean, all	146	611	20.40	7.20	0.00	22	0.22	nd	11	2.70	nd
	Australia and New Zealand	Lamb, all cuts, separable fat, raw	553	2315	10.80	57.60	0.00	34	0.07	0.07	5	0.41	0.5
	Australia and New Zealand	Mutton, all cuts, separable fat, raw	602	2522	8.20	64.40	0.00	64	0.07	2.90	10	0.77	1.0
	Western Africa	Lamb/mutton meat. lean. ca. 10% fat. raw	139	584	19.30	6.90	0.00	7	0.06	3.00	7	1.00	2.3
Average			360.00	1508.00	14.68	34.03	0.00	31.75	0.11	1.99	8.25	1.22	1.26
Standard deviation			251.96	1054.80	6.09	31.27	0.00	24.17	0.08	1.66	2.75	1.02	0.89
Goat	Australia and New Zealand	Goat, all cuts, separable fat, raw	443	1855	12.20	44.60	0.00	66	0.025	1.50	13	2.00	1.2
	USA	Game meat, goat, raw	109	456	20.60	2.31	0.00	0	0.49	1.13	13	2.83	4.0
	Western Africa	Goat lean raw	115	484	19.20	4.30	0.00	7	0.27	3.10	45	5.50	3.7
Average			222.33	931.67	17.33	17.07	0.00	24.33	0.38	2.30	23.67	3.75	3.83
Standard deviation			191.13	799.75	4.50	23.86	0.00	36.25	0.16	1.13	18.48	2.47	0.24
Pig	USA	Pork, fresh, separable fat, raw	632	2640	9.25	65.70	0.00	26	0.09	0.67	14	0.26	0.6
	Western Africa	Pork meat. lean. ca. 10% fat raw	152	634	18.60	8.60	0.00	2	0.02	0.30	31	0.10	0.2
Average			392.00	1637.00	13.93	37.15	0.00	14.00	0.06	0.49	22.50	0.18	0.42
Standard deviation			339.41	1418.46	6.61	40.38	0.00	16.97	0.05	0.26	12.02	0.11	0.26
Horse	USA	Game meat, horse, raw	133	556	21.4	4.60	0.00	0	0.10	3.00	6	3.82	2.9
Rabbit	ASEAN	Rabbit, whole carcass, raw	126	527	21.70	3.80	1.20	30	0.07	nd	16	1.30	nd
	Australia and New Zealand	Rabbit, farmed, whole, raw	113	472	23.20	2.10	0.00	10	0.06	5.3	9	1.00	1.6
	USA	Game meat, rabbit, domesticated, composite of cuts, raw	136	569	20.00	5.60	0.00	0	0.15	7.16	13	1.57	1.6
	Western Africa	Rabbit meat. raw	142	595	21.30	6.30	0.00	6	0.11	5.30	31	1.80	1.6
Average			130.33	545.33	21.50	4.67	0.00	3.00	0.09	6.23	17.67	1.40	1.60

Standard deviation			15.31	64.83	1.61	2.25	0.00	4.24	0.04	1.32	11.72	0.57	0.03
Deer	USA	Game meat, deer, raw	120	502	23.00	2.42	0.00	0	0.48	6.31	5	3.40	2.1
Chicken	USA	Poultry, mechanically deboned, from mature hens, raw	243	1020	14.70	20.00	0.00	45	0.14	0.27	187	1.22	1.9
	Western Africa	Chicken light meat withour skin, raw	116	491	23.00	2.70	0.00	8	0.08	0.23	14	1.10	0.7
Average			179.50	755.50	18.85	11.35	0.00	26.50	0.11	0.25	100.50	1.16	1.32
Standard deviation			89.80	374.06	5.87	12.23	0.00	26.16	0.04	0.03	122.33	0.08	0.82
Turkey	USA	Turkey, whole, meat only, raw	115	479	22.60	1.93	0.00	9	0.19	1.24	11	0.86	1.8
Quail	Australia and New Zealand	Quail, lean flesh & skin, raw	172	722	18.50	11.00	0.00	20	0.51	1.20	6	1.30	0.7
	USA	Quail, meat only, raw	134	561	21.80	4.50	0.00	17	0.29	0.47	13	4.51	2.7
Average			153.00	641.50	20.15	7.75	0.00	18.50	0.40	0.84	9.50	2.91	1.72
Standard deviation			26.87	113.84	2.33	4.60	0.00	2.12	0.16	0.52	4.95	2.27	1.39
Pheasant	USA	Pheasant, raw, meat only	133	556	23.60	3.60	0.00	50	0.15	0.84	13	1.15	1.0
Duck	ASEAN	Duck, meat, raw	233	975	15.60	19.00	0.00	nd	0.20	nd	14	1.80	nd
	Australia and New Zealand	Duck, lean flesh, raw	121	506	17.80	5.50	0.00	18	0.20	0.70	7	1.80	2.0
	USA	Duck, domesticated, meat only, raw	135	566	18.30	6.00	0.94	24	0.45	0.40	11	2.40	1.9
Average			131.69	682.33	17.23	10.17	0.31	21.00	0.28	0.55	10.67	2.00	1.95
Standard deviation			53.61	255.23	1.44	7.65	0.54	4.24	0.14	0.21	3.51	0.35	0.07
Goose	USA	Goose, domesticated, meat only, raw	161	674	22.80	7.10	0.00	12	0.38	0.49	13	2.57	2.34
Pigeon	Australia and New Zealand	Pigeon (squab), whole, raw	291	1219	16.20	25.50	0.00	73	0.22	0.40	13	2.40	2.2
	USA	Squab, (pigeon), meat only, raw	142	594	17.50	7.50	0.00	28	0.29	0.47	13	4.51	2.7
Average			216.50	906.50	16.85	16.50	0.00	50.50	0.25	0.44	13.00	3.46	2.45
Standard deviation			105.36	441.94	0.92	12.73	0.00	31.82	0.04	0.05	0.00	1.49	0.35
Guinea fow	USA	Guinea hen, meat only, raw	110	460	20.60	2.47	0.00	12	0.11	0.37	11	0.77	1.2
	Western Africa	Guinea fowl meat. raw	106	449	21.50	2.20	0.00	12	0.21	0.37	24	1.20	1.4
Average			108.00	454.50	21.05	2.34	0.00	12.00	0.16	0.37	17.50	0.99	1.29

Standard deviation	2.83	7.78	0.64	0.19	0.00	0.00	0.07	0.00	9.19	0.30	0.13
